# Magnetic resonance imaging-based classification of the myodural bridge complex and its influencing factors

**DOI:** 10.1007/s00276-023-03279-5

**Published:** 2024-01-09

**Authors:** Xiao Feng, Cong Liu, Dong-Mei Hu, Jian-Fei Zhang, Nan Zheng, Yan-Yan Chi, Sheng-Bo Yu, Hong-Jin Sui, Qiang Xu

**Affiliations:** 1 Department of Radiology, The 967 Hospital of the Joint Logistics Support Force, Dalian, 116021 China; 2 Department of Anatomy, College of Basic Medicine, Dalian Medical University, Dalian, 116044 China; 3 Postgraduate Training Base, The 967 Hospital of the Joint Logistics Support Force, Jinzhou Medical University, Dalian, 116021 China; 4 Department of Health Statistics, School of Public Health, Dalian Medical University, Dalian, 116044 China

**Keywords:** Myodural bridge complex, Imaging classification, Cervical spine, Degenerative changes, Magnetic resonance imaging, Dura mater, Cerebrospinal fluid

## Abstract

Cerebrospinal fluid (CSF) circulation is considered the third circulation of the human body. Recently, some scholars have proposed the myodural bridge (MDB) as a novel power source for CSF flow. Moreover, the suboccipital muscles can exert a driving force on the CSF via the MDB. This hypothesis is directly supported by head rotation and nodding movements, which can affect CSF circulation. The MDB has been validated as a normal structure in humans and mammals. In addition, the fusion of MDB fibers of different origins that act in concert with each other forms the MDB complex (MDBC). The MDBC may be associated with several CSF disorder-related neurological disorders in clinical practice. Therefore, the morphology of the MDBC and its influencing factors must be determined. In this study, T2-weighted imaging sagittal images of the cervical region were analyzed retrospectively in 1085 patients, and magnetic resonance imaging (MRI) typing of the MDBC was performed according to the imaging features of the MDBC in the posterior atlanto-occipital interspace (PAOiS) and posterior atlanto-axial interspace (PAAiS). The effects of age and age-related degenerative changes in the cervical spine on MRI staging of the MDBC were also determined. The results revealed four MRI types of the MDBC: type A (no MDBC hyposignal shadow connected to the dura mater in either the PAOiS or PAAiS), type B (MDBC hyposignal shadow connected to the dura mater in the PAOiS only), type C (MDBC hyposignal shadow connected to the dura mater in the PAAiS only), and type D (MDBC hyposignal shadow connected to the dura mater in both the PAOiS and PAAiS). The influencing factors for the MDBC typing were age (group), degree of intervertebral space stenosis, dorsal osteophytosis, and degenerative changes in the cervical spine (*P* < 0.05). With increasing age (10-year interval), the incidence of type B MDBC markedly decreased, whereas that of type A MDBC increased considerably. With the deepening of the degree of intervertebral space stenosis, the incidence of type C MDBC increased significantly, whereas that of type A MDBC decreased. In the presence of dorsal osteophytosis, the incidence of type C and D MDBCs significantly decreased, whereas that of type A increased. In the presence of protrusion of the intervertebral disc, the incidence of type B, C, and D MDBCs increased markedly, whereas that of type A MDBC decreased considerably, with cervical degenerative changes combined with spinal canal stenosis. Moreover, the incidence of both type C and D MDBCs increased, whereas that of type A MDBC decreased. Based on the MRI signal characteristics of the dural side of the MDBC, four types of the MDBC were identified. MDBC typing varies dynamically according to population distribution, depending on age and cervical degeneration (degree of intervertebral space stenosis, vertebral dorsal osteophytosis formation, simple protrusion of intervertebral disc, and cervical degeneration changes combined with spinal canal stenosis, except for the degree of protrusion of the intervertebral disc and the degree of spinal canal stenosis); however, it is not influenced by sex.

## Introduction

Cerebrospinal fluid (CSF) circulation is considered the third circulation of the human body with physiological significance. The CSF is contributed by arterial pulsation [10], ventricular diastolic activity [4, 6], ventricular choroid plexus dilation [29], postural changes [1], and ventricular morphology [2]. Sui et al. [28] proposed a new power source for the CSF for an inaugural time in the international arena, the myodural bridge (MDB). It may be a power source for CSF circulation; that is, the contraction of the suboccipital muscles may exert some driving force on the CSF through the connection between the MDB complex (MDBC) and spinal dura mater [26–28]. Head movements affect CSF flow, adding a direct line of evidence to this theory [22, 23]. Neurological disorders, such as Chiari malformation and syringomyelia, are associated with CSF circulation [7, 13], and the MDBC may play an essential role in the development and progression of these disorders. Therefore, MDBC morphology and its influencing factors should be explored.

The MDBC fibers connecting the dura mater through the posterior atlanto-occipital interspace (PAOiS) are derived from the rectus capitis posterior minor muscle (RCPmi) and rectus capitis posterior major muscle (RCPma). The MDBC fibers connecting the dura through the posterior atlanto-axial interspace (PAAiS) originate from the RCPmi, RCPma, and obliquus capitis inferior (OCI) muscle. The MDBC acts on the dura mater through the PAOiS and PAAiS. The MDBC, a composite structure, is formed by the fusion of MDB fibers of different origins that act in concert with each other [27].

Studies on the magnetic resonance imaging (MRI) of the MDB have been conducted, and MRI-based MDB morphological studies have been deemed feasible [8, 15–17]. Degenerative disc changes and spinal canal stenosis occur to varying degrees in the cervical spine during the aging process [12, 14, 20]. Morphological typing of MDBC and its modifications with age and degenerative changes in the cervical spine have not yet been reported. In this study, the imaging classification of the MDBC was classified using MRI. Moreover, the influence of sex, age, and degenerative changes of the cervical spine on MDBC classification was retrospectively analyzed in a large sample to provide an imaging basis for functional studies and clinical applications of the MDBC.

## Materials and methods

### Study participants

The approval of the institutional review board was obtained and written informed consent was waived due to the retrospective nature of the study.Imaging data from adult participants who underwent neck MRI with routine examination findings (both normal cervical and age-related cervical degeneration) at our hospital between January 2017 and January 2022 were selected for retrospective analysis. The inclusion criteria were as follows: (1) patients aged 20–69 years, (2) without history of neck surgery, and (3) without head and neck deformities, trauma, or other conditions that may affect the structure of the head and neck. Additionally, the results of the first test were obtained for multi-trial candidates. Patients with incomplete imaging data were excluded from this study. Finally, 1085 (518 men and 567 women; mean age, 43.3 ± 13.6 years) susceptible individuals (including those with degenerative cervical spine) were included. The patients were divided according to age into the 20–29-, 30–39-, 40–49-, 50–59-, and 60–69-year-old groups.

### Study methods

#### Magnetic resonance imaging (MRI) scanning

MRI was performed using a 1.5-T scanner (Signa, HDxt, GE Healthcare, USA). The participants were scanned in the supine position using a cervical phased-array coil covering the occipital and suboccipital regions. The sagittal parameters of T2-weighted imaging (T2WI) imaging were as follows: repetition time, 2740.0 ms; echo time, 120.0 ms; number of layers, 11; layer thickness, 3 mm; matrix, 256 × 256; and field of view, 240 × 240 mm.

#### Criteria for the assessment of degenerative changes in the cervical spine

The criteria for selecting the indicators of cervical degenerative changes were as follows: Based on the findings by Fakhoury et al. [5], the four common degenerative changes in the cervical spine, namely, disc herniation, spinal stenosis, intervertebral space narrowing, and dorsal bone redundancy, were selected for assessment in this study.

The assessment criteria for the indicators of cervical degenerative changes were as follows: C2–T1 cervical degenerative changes were assessed based on the MRI classification system adopted by Wang et al.[21] and Daimon et al.[3] (Table 1). The presence and severity of degenerative changes in the six cervical vertebral segments were recorded for each participant, and only the most severe cases were considered for each cervical degenerative change. Coexistence of degenerative changes in the cervical spine was also observed. The MRI readings were performed by two radiologists. MRI images were evaluated separately by both readers, and the results were compared. In cases in which the assessments differed, the MRI images were read again and agreed upon.

**Table 1:** Magnetic resonance imaging-based classification system for the indicators of degenerative changes in the cervical spine

Protrusion of the intervertebral disc
Grade 0: No herniated disc
Grade 1: Herniated disc beyond the posterior border of the vertebral body without spinal cord compression
Grade 2: Herniated disc beyond the posterior border of the vertebral body with spinal cord compression
Spinal canal stenosis
Grade 0: Normal or mild subarachnoid stenosis
Grade 1: Anterior subarachnoid stenosis ≥ 50% without spinal cord compression
Grade 2: Spinal stenosis ≥ 50% with spinal cord compression
Stenosis of the intervertebral space
Grade 0: > 75% upper healthy disc height
Grade 1: 50%–75% upper healthy disc height
Grade 2: < 50% upper healthy disc height
Dorsal osteophytosis formation
Grade 0: None
Grade 1: Evident dorsal osteophytosis

#### MRI assessment criteria for myodural bridge complex (MDBC) staging

On T2WI images in the sagittal position, the MDBC was a linear or cord-like low-signal shadow originating from the suboccipital muscle, nuchal ligament, and/or cervical vertebral body, passing through the PAOiS and/or PAAiS, and eventually connected to the dura mater of the upper cervical spine. The PAOiS and PAAiS were observed for low-signal linear or cord-like hyposignal shadows in both spaces that were connected to the dura mater. Imaging classification of the MDBC was performed according to the location of the MDBC hyposignal shadow with the dura mater.

The imaging data of the 1085 enrolled participants were provided to two radiologists (both with experience in MDBC-related studies, who had been trained in typing methods), and the typing results were recorded. One month later, the original 1085 enrolled participants were renumbered and then typed again using the same method, and the MDBC typing of the participants in the dispute was finally determined by two observers after consultation.

Cautions for the typing process were as follows: (1) The typing process was independently completed by two observers independently; (2) basic data of the participants were concealed, and only cervical MRI images of the participants were available; and (3) the double-blind principle was adopted for both types.

### Statistical analyses

The Statistical Package for the Social Sciences (SPSS) version 26.0 (SPSS Inc., Chicago, IL, USA) was used for statistical analyses. Frequencies and rates were used to express counting data. Cohen’s kappa coefficient and mean kappa coefficient values were calculated to assess the MDBC imaging classification and concordance of two observations from two observers. Kappa values of 0–0.20, 0.21–0.40, 0.41–0.60, 0.61–0.80, and 0.81–1 were considered poor, fair, moderate, good, and strong consistencies, respectively. The chi-squared test was used for univariate analysis, and a multi-factor logistic stepwise regression analysis was performed. The inspection level was set at* α* = 0.05. Statistical significance was set at *P* < 0.05.

## Results

### The MDBC imaging classification

The MDBC was classified into four types, A, B, C, and D, based on the signal characteristics of the location of the MDBC connection to the dura mater (PAOiS, PAAiS).

**Fig. 1:**
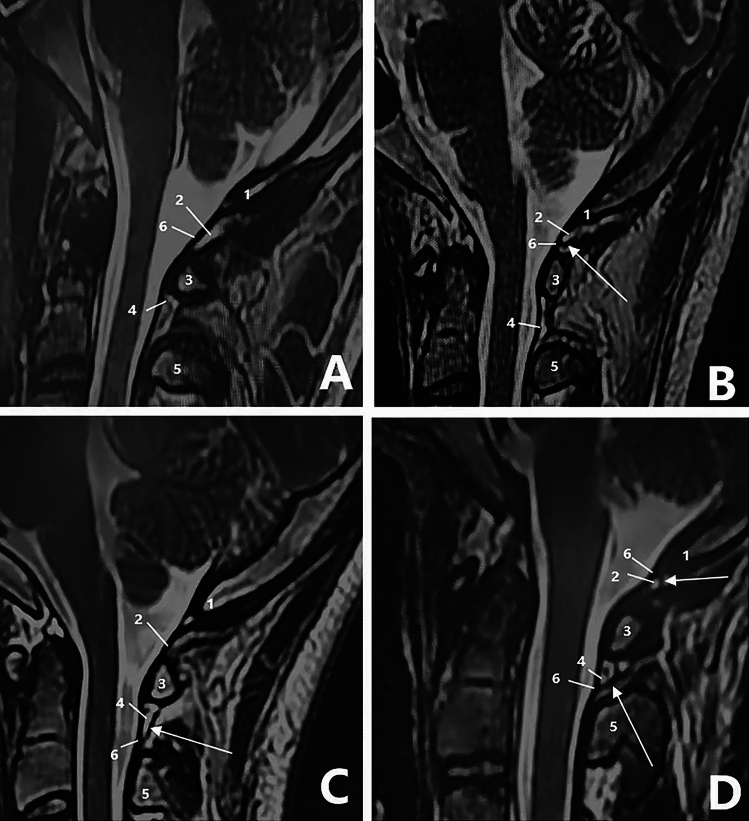
The T2-weighted imaging image of the occipital and cervical portions (sagittal view) 1, occipital bone; 2, posterior atlanto-occipital interspace (PAOiS); 3, posterior arch of atlas; 4, posterior atlanto-axial interspace (PAAiS); 5, spinous process of axis; 6, upper cervical dura mater. Arrows indicate the myodural bridge (MDB). Figure **A**: Type A MDB complex (MDBC): no MDBC low-signal shadow connected to the dura mater in either the PAOiS or PAAiS, Figure **B**: Type B MDBC: MDBC low-signal shadow connected to the dura mater in the PAOiS only (arrow), Figure **C**: Type C MDBC: MDBC low-signal shadow connected to the dura mater in the PAAiS only (arrow), and Figure **D**: Type D MDBC: both the PAOiS and PAAiS show MDBC hyposignal shadow with dural connection (arrow)

Type A MDBC: No MDBC hyposignal shadow is connected to the dura in either the PAOiS or PAAiS, with a fatty hypersignal in both spaces and some vascular flow in the space (Fig. 1A).

Type B MDBC: Only the PAOiS has an MDBC hyposignal shadow connected to the dura mater, whereas the PAAiS has a fatty hypersignal and some vascular flow (Fig. 1B).

Type C MDBC: Only the PAAiS shows an MDBC hyposignal shadow connected to the dura mater, with a fatty high signal in the PAOiS (Fig. 1C).

Type D MDBC: The PAOiS and PAAiS both show MDBC hyposignal shadows connected to the SDM (Fig. 1D).

In total, 499 (46.0%), 213 (19.6%), 208 (19.2%), and 165 (15.2%) patients had type A, B, C, and D MDBC, respectively (Table 2).

**Table 2:** Results of myodural bridge complex (MDBC) imaging classification

Type	Type A MDBC	Type B MDBC	Type C MDBC	Type D MDBC
Number of patients (patients [%])	499 (46.0)	213 (19.6)	208 (19.2)	165 (15.2)

### Consistency analysis of MDBC classification

The classification method was used to image 1,085 subjects twice by the same observer, and the Kappa concordance results indicated a high level of consistency in retesting (Kappa = 0.984).

Two observers used this classification method to type 1085 subjects, and the Kappa concordance results showed a very high level of agreement between them (Kappa = 0.873). The concordance rates were 88.38% (441/499) for type A MDBC, 92.96% (198/213) for type B MDBC, 91.83% (191/208) for type C MDBC, and 95.76% (158/165) for type D MDBC.

The inconsistency rate between the two observers was 11.62% for type A MDBC, with 29 cases of type B MDBC, 25 cases of type C MDBC, and 4 cases of type D MDBC. For type B MDBC, the inconsistency rate was 7.04%, with 8 cases of type A MDBC and 7 cases of type D MDBC. For Type C MDBC, the inconsistency rate was 8.17%, with 4 cases of Type A MDBC and 13 cases of Type D MDBC. Finally, for Type D MBCD, the inconsistency rate was 4.24%; one case was classified as Type A, two were identified as Type B, and another four were categorized as Type C.

### Univariate analysis of factors influencing MDBC imaging classification

The results showed that the MRI typing of the MDBC was influenced by sex, age, degree of intervertebral space stenosis, dorsal osteophytosis, and a combination of degenerative changes in the cervical spine (*P* < 0.05), but not by the degree of protrusion of the intervertebral disc and spinal canal stenosis (*P* > 0.05) (Tables 3, 4, and 5).

**Table 3:** Univariate analysis of the influence of general data on the myodural bridge complex (MDBC) types

Influencing factors	Type A MDBC (patients [%])	Type B MDBC (patients [%])	Type C MDBC (patients [%])	Type D MDBC (patients [%])	χ^2^	*P*
*Sex*						
Male	220 (44.1)	103 (48.4)	98 (47.1)	97 (58.8)	10.804	0.013^*^
Female	279 (55.9)	110 (51.6)	110 (52.9)	68 (41.2)		
*Age *(group)						
20–29	73 (36.5)	39 (19.5)	49 (24.5)	39 (19.5)	29.551	0.003^*^
30–39	119 (42.8)	60 (21.6)	47 (16.9)	52 (18.7)		
40–49	102 (46.2)	48 (21.7)	42 (19.0)	29 (13.1)		
50–59	105 (50.0)	35 (16.7)	37 (17.6)	33 (15.7)		
60–69	100 (56.8)	31 (17.6)	33 (18.8)	12 (6.8)		

*Statistically significant difference (*P* < 0.05)

**Table 4:** Univariate analysis of the influence of degenerative changes in the cervical spine on the myodural bridge complex (MDBC) types

Indicators	Type A MDBC (patients [%])	Type B MDBC (patients [%])	Type C MDBC (patients [%])	Type D MDBC (patients [%])	χ^2^	*P*
*Degree of intervertebral disc protrusion*						
Grade 0	105 (48.4)	38 (17.5)	39 (18.0)	35 (16.1)	2.713	0.844
Grade 1	294 (44.5)	136 (20.6)	133 (20.1)	98 (14.8)		
Grade 2	100 (48.3)	39 (18.8)	36 (17.4)	32 (15.5)		
*Degree of spinal canal stenosis*						
Grade 0	243 (44.0)	115 (20.8)	108 (19.6)	86 (15.6)	2.599	0.857
Grade 1	157 (47.9)	61 (18.6)	64 (19.5)	46 (14.0)		
Grade 2	99 (48.3)	37 (18.0)	36 (17.6)	33 (16.1)		
*Degree of intervertebral disc narrowing*						
Grade 0	231 (40.3)	118 (20.6)	111 (19.4)	113 (19.7)	30.702	0^*^
Grade 1	116 (55.0)	51 (16.9)	50 (16.6)	35 (11.6)		
Grade 2	102 (48.6)	44 (21.0)	47 (22.4)	17 (8.1)		
*Dorsal osteophytosis formation*						
Grade 0	185 (37.5)	89 (18.1)	113 (22.9)	106 (21.5)	45.390	0^*^
Grade 1	314 (53.0)	124 (20.9)	95 (16.0)	59 (10.0)		

*Statistically significant difference with *P* < 0.05

**Table 5:** Univariate analysis of the influence of multiple cervical degenerative changes acting together on MDBC types

Indicators	Type A MDBC (patients [%])	Type B MDBC (patients [%])	Type C MDBC (patients [%])	Type D MDBC (patients [%])	χ^2^	*P*
*Joint action of multiple degenerative changes*						
Normal cervical vertebra	105 (48.4)	38 (17.5)	39 (18.0)	35 (16.1)	110.079	0^*^
Protrusion of the intervertebral disc only	47 (27.3)	41 (23.8)	46 (26.7)	38 (22.1)		
Protrusion of the intervertebral disc + spinal canal stenosis	17 (27.0)	6 (9.5)	13 (20.6)	27 (42.9)		
Protrusion of the intervertebral disc + intervertebral space stenosis	8 (42.1)	2 (10.5)	5 (26.3)	4 (21.1)		
Protrusion of the intervertebral disc + dorsal osteophytosis	25 (48.1)	15 (28.8)	7 (13.5)	5 (9.6)		
Protrusion of the intervertebral disc + spinal canal stenosis + intervertebral space stenosis	8 (34.8)	3 (13.0)	10 (43.5)	2 (8.7)		
Protrusion of the intervertebral disc + spinal canal stenosis + dorsal osteophytosis	37 (53.6)	18 (26.1)	6 (8.7)	8 (11.6)		
Protrusion of the intervertebral disc + intervertebral space stenosis + dorsal osteophytosis	58 (63.0)	19 (20.7)	11 (12.0)	4 (4.3)		
Protrusion of the intervertebral disc + spinal canal stenosis + intervertebral space stenosis + dorsal osteophytosis	194 (51.3)	71 (18.8)	71 (18.8)	42 (11.1)		

*Statistically significant difference at *P* < 0.05

### Multi-factor analysis of factors influencing MDBC imaging classification

The sex, age (group), degree of intervertebral disc protrusion, degree of spinal canal stenosis, degree of intervertebral space stenosis, osteophytosis formation, and joint action of multiple degenerative changes were used as independent variables, and MDBC MRI typing was used as the dependent variable. A multifactorial logistic regression model with type A MDBC as the reference was developed. Fakhoury et al. [5] concluded that in the process of degenerative changes in the human cervical spine, intervertebral disc protrusion was a result of the dysfunctional and unstable phases, whereas spinal canal stenosis was caused by osteophytosis and intervertebral space narrowing. Therefore, in this study, participants with multiple degenerative changes combined with or without protrusion of the intervertebral disc or spinal canal stenosis were divided into four groups: normal cervical spine, protrusion of the intervertebral disc, degenerative changes in the cervical spine (protrusion of the intervertebral disc, intervertebral space stenosis, and dorsal osteophytosis) without spinal canal stenosis, and degenerative changes in the cervical spine (protrusion of the intervertebral disc, intervertebral space stenosis, and dorsal osteophytosis) with spinal canal stenosis.

The factors influencing MDBC imaging typing were age (group), degree of intervertebral space stenosis, osteophytosis formation, and the combined action of multiple degenerative changes based on multifactorial analysis (*P* < 0.05).

The incidence of type B and A MDBCs decreased and increased, respectively, with increasing age (10 years per age group), and the probability of type B MDBC decreased to 0.977 times that of type A MDBC with each 10-year increase in age (odds ratio [OR] = 0.977, *P* < 0.05).

There was an increase in incidence of type C MDBC and a decrease in the incidence of type A MDBC with the progression of cervical intervertebral space stenosis, and the probability of type C MDBC increased 1.586 times that of type A MDBC for each grade of severity of intervertebral space stenosis (OR = 1.586, *P* < 0.05).

Following the appearance of dorsal osteophytosis, there was a decrease in the incidence of type C and D MDBCs and an increase in the incidence of type A MDBC. The probability of type C and D MDBCs decreased to 0.296 and 0.297 times that of type A MDBC, respectively (OR = 0.296 and 0.297, respectively;* P* < 0.05).

In the protrusion of the cervical intervertebral disc, compared to the normal cervical vertebra, the incidence of type B, C, and D MDBCs increased, whereas that of type A MDBC decreased. The odds of type B, C, and D MDBC occurrence increased to 2.760, 2.821, and 2.582 times that of type A MDBC, respectively (OR = 2.760, 2.821, and 2.582, respectively; *P* < 0.05).

In the case of cervical degenerative changes combined with spinal canal stenosis, there was an increase in the incidence of type C and D MDBC and a decrease in the incidence of type A MDBC when compared with normal cervical vertebrae. The incidence of type C and D MDBCs increased to 2.207 and 4.112 times that of type A MDBC, respectively (OR = 2.207 and 4.112, respectively; *P* < 0.05) (Table 6).

**Table 6:** Multi-factor logistic regression analysis of factors influencing MDBC imaging typing

Typing	Factors	B	*P*	OR	OR 95% CI	
Type B MDBC	Age (group)	– 0.023	0.011*	0.977	0.959	0.995
	Degree of intervertebral space stenosis	0.040	0.793	1.041	0.769	1.409
	Formation of dorsal osteophytosis	0.501	0.196	1.651	0.772	3.530
	Joint action of multiple degenerative changes					
	Protrusion of the intervertebral disc only	1.015	0*	2.760	1.561	4.880
	Cervical degenerative changes with no spinal canal stenosis	0.073	0.869	1.076	0.450	2.572
	Cervical degenerative changes with spinal canal stenosis	0.076	0.856	1.078	0.477	2.437
	Normal cervical vertebra	0				
Type C MDBC	Age (group)	– 0.012	0.212	0.988	0.969	1.007
	Degree of intervertebral space stenosis	0.462	0.006*	1.586	1.141	2.206
	Dorsal osteophytosis formation	– 1.216	0*	0.296	0.158	0.555
	Joint action of multiple degenerative changes					
	Protrusion of the intervertebral disc only	1.037	0*	2.821	1.611	4.939
	Cervical degenerative changes with no spinal canal stenosis	0.464	0.237	1.590	0.737	3.431
	Cervical degenerative changes with spinal canal stenosis	0.792	0.022*	2.207	1.123	4.339
	Normal cervical vertebra	0				
Type D MDBC	Age (group)	– 0.011	0.295	0.989	0.969	1.010
	Degree of intervertebral space stenosis	– 0.280	0.136	0.756	0.523	1.092
	Dorsal osteophytosis formation	– 1.276	0*	0.279	0.148	0.527
	Joint action of multiple degenerative changes					
	Protrusion of the intervertebral disc only	0.949	0.002*	2.582	1.435	4.645
	Cervical degenerative changes with no spinal canal stenosis	0.669	0.126	1.953	0.829	4.603
	Cervical degenerative changes with spinal canal stenosis	1.414	0*	4.112	2.118	7.983
	Normal cervical vertebra	0				

*Statistically significant difference at *P* < 0.05

## Discussion

### MDBC imaging classification methods

Previously, the morphology of MDB via MRI demonstrated the feasibility of morphological studies of MDB based on MRI techniques [8, 15–17]. In this study, an MRI-based classification of the MDBC was developed, with the typical T2WI signal presenting as a low-signal band from the suboccipital muscle, nuchal ligament, or vertebral body ending at the dura mater through the PAOiS or PAAiS. Moreover, depending on where the low signal was attached to the dura mater (PAOiS, PAAiS), the MDBC was classified into four types: A, B, C, and D. The distribution of the four types of the MDBC among the 1085 participants was statistically analyzed. Type A, B, C, and D MDBCs accounted for 46.0%, 19.6%, 19.2%, and 15.2% of the participants, respectively.

In this study, the MDBC was simultaneously observed in the PAOiS and PAAiS for the first time. Previous studies have focused on the morphology of the MDBC in the PAOiS or PAAiS alone; however, it has not been described and studied as a functional whole, concurrently with multiple sources of the MDBC in the suboccipital region. Humphreys et al. [8] in a controlled study of anatomical specimens and magnetic resonance images showed that the connection between the RCPmi and dura mater was displayed on T1-weighted imaging as a low-signal band from the RCPmi across the PAOiS toward the dura mater. Moreover, this low-signal band was part of the imaging presentation of the MDBC in the PAOiS, which was proposed as type B MDBC or part of type D MDBC in this study. Scali et al. [15] and Shao et al. [16] described a low-signal shadow of the MDB in the PAAiS connected to the dura mater, a part of the MDBC in the PAAiS, which was proposed as type C MDBC or part of type D MDBC for this study. Additionally, Sun et al. [17] characterized the connection between the to be named ligament (TBNL) of the PAAiS and RCPmi on T2WI images using three-dimensional (3D) MRI, where the TBNL was involved in forming the MDBC in the PAAiS. The TBNL described in this study was also a manifestation of the PAAiS of the type C MDBC or part of type D MDBC in this study.

**Fig. 2:**
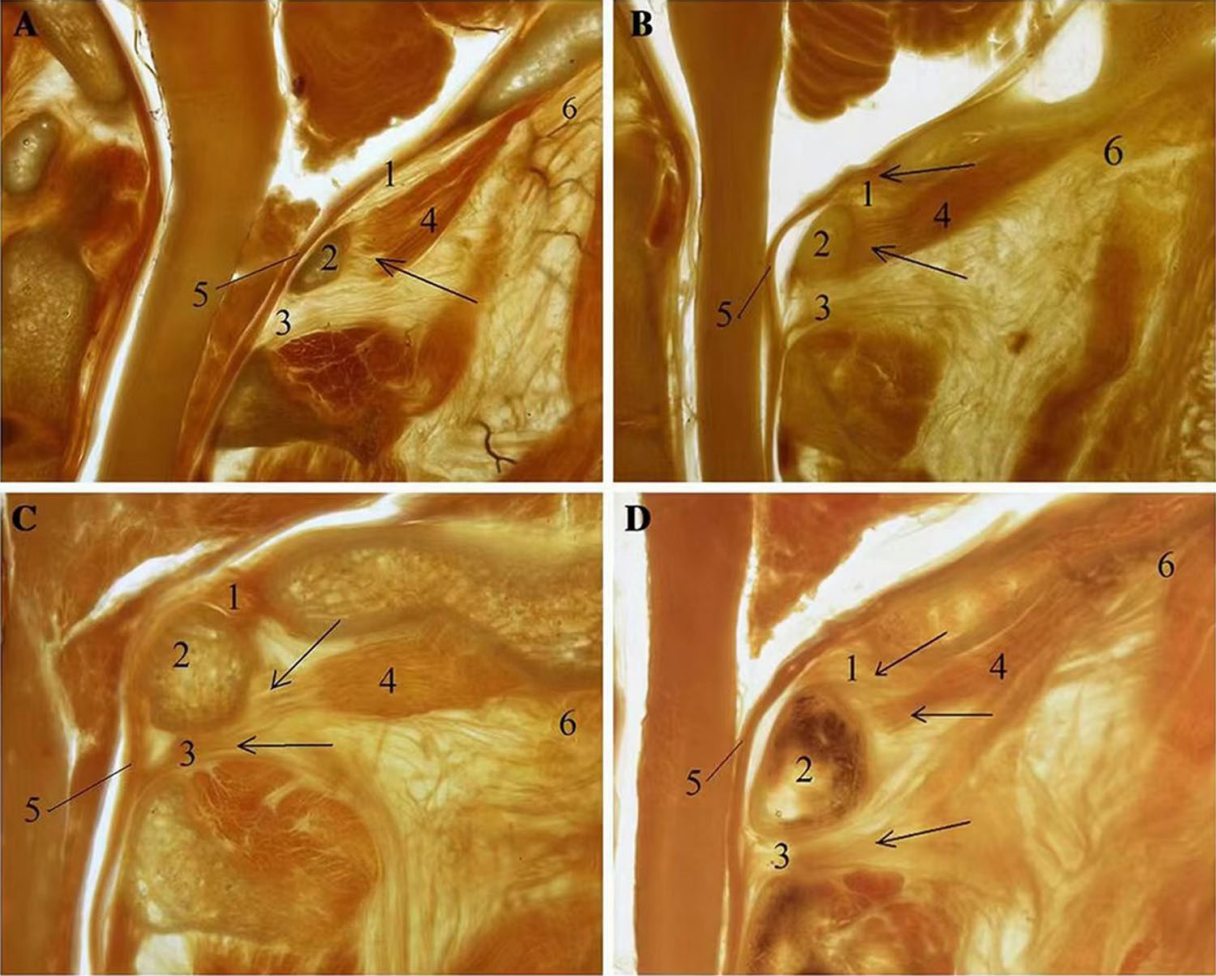
P45 plasticized specimen in the suboccipital region (midsagittal images) (Yuan et al. [25]). Figure **A**: Sparse and scattered myodural bridge (MDB) fibers in the posterior atlanto-occipital interspace (PAOiS) and posterior atlanto-axial interspace (PAAiS), consistent with type A MDB complex (MDBC), and Figures **B**, **C**, and **D**: MDB fibers aggregated in bundles in the PAOiS and PAAiS, consistent with type D MDBC. 1, PAOiS; 2, posterior arch of atlas; 3, PAAiS; 4, rectus capitis posterior minor muscle; 5, upper cervical dura; and 6, inferior nuchal line

Previous anatomical studies have identified the MDBC as concurrently present in the PAOiS and PAAiS. Zheng et al. discovered that the MDBC coexisted with the MDB originating from the RCPmi, RCPma, and OCI muscle [27]. A portion of the MDB originates from the ventral side of the RCPmi and travels with the cranial segment of the RCPma through the PAOiS to connect with the dura mater. Another portion of the MDB fibers emanated from the dorsal aspect of the RCPmi and the ventral aspect of the caudal segment of the RCPma, and the ventral aspect of the medial segment of the OCI muscle entered the central portion of the PAAiS and fused with the vertebral dural ligament. In 2016, Yuan et al. [25] found that the RCPmi was connected to the PAAiS in addition to the PAOiS and dura mater through P45 plastinated specimens combined with gross anatomy. In addition, they proposed four termination types of RCPmi, suggesting the morphological diversity of the suboccipital group. Their reported findings also offered an anatomical basis for partial imaging typing of the MBDC proposed in this study (Fig. 2). The results of this study, in conjunction with previous anatomical studies of the MDBC, enabled us to conclude that the MBDC was morphologically diverse.

### Factors influencing the MRI typing of the MDBC

In this study, age and cervical degeneration substantially contributed to MDBC imaging staging.

The population distribution of MDBC imaging classification changed noticeably with age. The analysis indicated a decrease and increase in the incidence of type B and C MDBC, respectively, with aging. Additionally, the present study concluded that sex did not have a noticeable effect on MDBC typing.

Among the indicators of cervical degeneration included in the examination, the incidence of type A MDBC increased in the presence of dorsal osteophytosis, whereas the incidence of both type C and D MDBC decreased. Aging and dorsal osteophytosis formation share similarities in their influence on the population distribution of MDBC imaging classification, and additional studies are warranted on their influence on MDBC type and whether they share common mechanisms.

The degree of intervertebral space stenosis is another factor affecting MDBC typing. The degree of intervertebral space stenosis intensifies, with more type C MDBC and fewer type A MDBC cases. A decrease in cervical disc height has been demonstrated to possibly lead to cervical instability and normal cervical curvature loss. In all changes in the cervical curvature, there is a compensatory C0–2 angle to maintain a normal horizontally oriented view, with the RCPmi and RCPma playing a critical role in this process. This may be associated with an increase in the incidence of type C MDBC and a decrease in the incidence of type A MDBC [11, 19, 24].

### The potential of MRI techniques in MDBC research

MRI techniques are widely used in clinical studies related to MDBC, providing both direct anatomical information and functional insights. Phase-contrast magnetic resonance imaging (PC-MRI) is an important tool for assessing cerebrospinal fluid circulation dynamics. By using PC-MRI techniques, Xu et al. [22, 23] discovered that head shaking and nodding motions dramatically altered the cerebrospinal fluid. Head movements further influenced the cerebrospinal fluid circulation. This provided strong evidence for the involvement of the MDBC in the regulation of CSF circulation. On the other hand, new methods are being utilized more and more to study the cerebrospinal fluid circulation, such as 4D Flow MRI, which can show the cerebrospinal fluid moving in several directions and has a greater spatial resolution that makes it easier to quantify pressure gradients. By pulling on the dura mater through the MDBC and altering the morphology of the subarachnoid space in the corresponding region, the suboccipital muscles’ involvement in head movements further influenced the cerebrospinal fluid circulation. This provided strong evidence for the involvement of the MDBC in the regulation of cerebrospinal fluid circulation. On the other hand, new methods are being utilized more and more to study the cerebrospinal fluid circulation, such as 4D Flow MRI, which can show the cerebrospinal fluid moving in several directions and has a greater spatial resolution that makes it easier to quantify pressure gradients. Heidari et al. [9] assessed the accuracy of 4D Flow MRI for cerebrospinal fluid dynamics and discovered that it was a reliable assessment of cerebrospinal fluid flow rate and distribution. Furthermore, MRI techniques can provide functional information on MDBC-related muscles, allowing for a more specific assessment of MDBC, which requires certain special sequences or MRI techniques, such as magnetic resonance spectroscopy (MRS), diffusion-weighted imaging (DWI), diffusion tensor imaging (DTI), and so on [18]. The more extensive examination of MDBC-related muscle tissue allows for a better understanding of the pathophysiology of MDBC-related illness and the development of a more detailed treatment strategy. Unlike traditional MRI scans, which only give anatomical information, the use of functional MRI imaging will uncover problems in some ‘normal’ structures, which will be a new trend in future functional research connected to MDBC.

## Concluding remarks

Based on the MRI signal characteristics of the PAOiS and PAAiS, the MDBC consists of four imaging types, and the morphology of MDBC features population diversity.

MDBC MRI typing is influenced by age and degenerative changes in the cervical spine, and MDBC imaging classification is distributed variably in the population.

## Limitations of this study

The limitations of this study are as follows: (1) This was a single-center retrospective study. (2) Owing to the limitations of conventional MRI in terms of clarity and layer thickness, some false-negative results for the MDBC may have resulted in the increased incidence of type A MDBC in this study. This may be due to the application of high-resolution or 3D MRI. (3) The present study failed to quantify the imaging indicators of the MDBC, and subsequent studies should be conducted to adopt special MRI sequences and corresponding post-processing techniques to quantify the MDBC. (4) The population selected in the present study did not include individuals aged < 20 and ≥ 70 years, and some limitations may be present in the description of the distribution of MDBC types in the entire population. (5) As this was not a natural history study on cervical degenerative changes, only the four most common and significant cervical degenerative changes were selected to determine the cervical degenerative changes without incorporating other degenerative changes. Our results highlight that degenerative changes in the cervical spine might influence the MDBC type via some mechanisms. However, further studies are required to confirm the specific changes and mechanisms of influence of MDBC type throughout the progression of degenerative changes in the cervical spine.

## Data Availability

The raw data supporting the findings of this study are available from the corresponding authors upon reasonable request.
